# The Binding of *Alpinia galanga* Oil and Its Nanoemulsion to Mammal GABA_A_ Receptors Using Rat Cortical Membranes and an In Silico Modeling Platform

**DOI:** 10.3390/pharmaceutics14030650

**Published:** 2022-03-16

**Authors:** Nattakanwadee Khumpirapang, Krit Suknuntha, Pathomwat Wongrattanakamon, Supat Jiranusornkul, Songyot Anuchapreeda, Petrine Wellendorph, Anette Müllertz, Thomas Rades, Siriporn Okonogi

**Affiliations:** 1 Department of Pharmaceutical Chemistry and Pharmacognosy, Faculty of Pharmaceutical Sciences, Naresuan University, Phitsanulok 65000, Thailand; nattakanwadeek@nu.ac.th; 2 Department of Pharmaceutical Chemistry, Faculty of Pharmaceutical Sciences, Prince of Songkla University, Songkhla 90112, Thailand; krit@pharmacy.psu.ac.th; 3 Department of Pharmaceutical Sciences, Faculty of Pharmacy, Chiang Mai University, Chiang Mai 50200, Thailand; pathomwat.w@cmu.ac.th (P.W.); supat.jira@cmu.ac.th (S.J.); 4 Department of Medical Technology, Faculty of Associated Medical Sciences, Chiang Mai University, Chiang Mai 50200, Thailand; songyot.anuch@cmu.ac.th; 5 Research Center of Pharmaceutical Nanotechnology, Faculty of Pharmacy, Chiang Mai University, Chiang Mai 50200, Thailand; 6 Department of Drug Design and Pharmacology, Faculty of Health and Medical Sciences, University of Copenhagen, 2100 Copenhagen, Denmark; pw@sund.ku.dk; 7 Department of Pharmacy, Faculty of Health and Medical Sciences, University of Copenhagen, 2100 Copenhagen, Denmark; anette.mullertz@sund.ku.dk (A.M.); thomas.rades@sund.ku.dk (T.R.)

**Keywords:** *Alpinia galanga*, essential oil, mammal anesthesia, anesthetic pathway, positive allosteric modulation, binding assay

## Abstract

The anesthetic effect of *Alpinia galanga* oil (AGO) has been reported. However, knowledge of its pathway in mammals is limited. In the present study, the binding of AGO and its key compounds, methyl eugenol, 1,8-cineole, and 4-allylphenyl acetate, to gamma-aminobutyric acid type A (GABA_A_) receptors in rat cortical membranes, was investigated using a [^3^H]muscimol binding assay and an in silico modeling platform. The results showed that only AGO and methyl eugenol displayed a positive modulation at the highest concentrations, whereas 1,8-cineole and 4-allylphenyl acetate were inactive. The result of AGO correlated well to the amount of methyl eugenol in AGO. Computational docking and dynamics simulations into the GABA_A_ receptor complex model (PDB: 6X3T) showed the stable structure of the GABA_A_ receptor–methyl eugenol complex with the lowest binding energy of −22.16 kcal/mol. This result shows that the anesthetic activity of AGO and methyl eugenol in mammals is associated with GABA_A_ receptor modulation. An oil-in-water nanoemulsion containing 20% w/w AGO (NE-AGO) was formulated. NE-AGO showed a significant increase in specific [^3^H]muscimol binding, to 179% of the control, with an EC_50_ of 391 µg/mL. Intracellular studies show that normal human cells are highly tolerant to AGO and the nanoemulsion, indicating that NE-AGO may be useful for human anesthesia.

## 1. Introduction

*Alpinia galanga*, an edible plant of the Zingiberaceae family, is widely cultivated in Southeast Asian countries [1]. It is well-known in Asian folk medicine and has been used for centuries as a food additive, an antimicrobial agent, a local anesthetic, an analgesic, and an antipruritic [2,3]. Three important compounds, methyl eugenol, 1,8-cineole, and 4-allylphenyl acetate, were reported in *A. galanga* oil (AGO) [4]. Recently, AGO has been shown to have an anesthetic effect in fish [5]. Further, 1,8-cineole has been shown to reduce locomotor activity in rodents when administered orally or intraperitoneally [6,7]. To the best of our knowledge, the mechanism of the anesthetic action of AGO has not yet been elucidated. Moreover, 4-allylphenyl acetate has not been reported for anesthetic effect in rodents, whereas methyl eugenol, a minor compound of AGO, has been reported as a surgical anesthetic in rodents [8].

**Figure 1:**
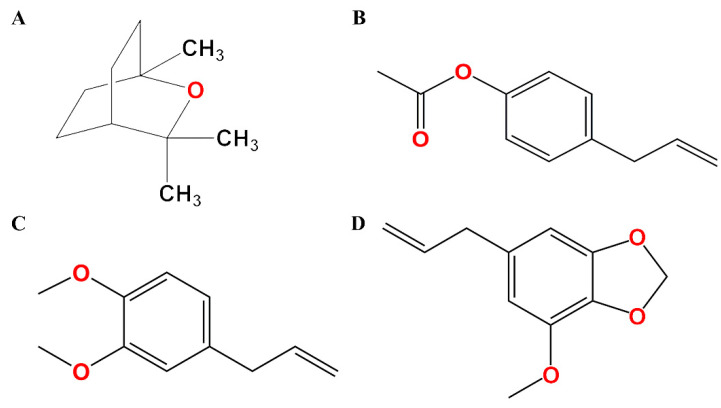
Chemical structures of (**A**) 1,8-cineole, (**B**) 4-allylphenyl acetate, (**C**) methyl eugenol, and (**D**) myristicin.

Generally, anesthetics have been used to relieve pain and suffering during surgery and post-surgery by inhibiting or depressing the propagation of the pain signal along the nerves [9]. The mechanisms of action of the anesthetics include the blockade of the N-methyl-D-aspartate receptors, the inhibition of dopaminergic receptors, and the enhancement of the function of γ-aminobutyric acid type A (GABA_A_) receptors [10]. GABA_A_ receptors are the principal ionotropic receptors for fast inhibitory neurotransmission in the mammalian central nervous system [11]. GABA_A_ receptors are clinically employed targets for a range of structurally diverse positive allosteric modulators, such as isoflurane, etomidate, propofol, barbiturates, and benzodiazepines [12]. As seen in Figure 1, among the three important compounds of AGO mentioned above, the chemical structures of 4-allylphenyl acetate and methyl eugenol are similar to myristicin, a previously reported GABA_A_ receptor of positive allosteric modulators [13]. Therefore, we hypothesized that the mechanism of the anesthetic action of AGO may involve central GABA_A_ receptors.

The poor water solubility of active compounds in essential oils remains a challenging problem in medical applications and bioanalysis studies using aqueous buffers. To overcome this problem, various organic solvents are used for dissolving or delivering these active compounds. However, those organic solvents may affect the biological assessment and application [14]. AGO is immiscible with water and it requires a potential system for water-miscible enhancement. Nanoemulsion is one of the promising delivery systems that can improve the aqueous solubility of many hydrophobic active compounds [15,16,17]. Several nanoemulsions of water-insoluble compounds have been developed for these applications [18,19,20,21].

In the present study, the chemical compositions of AGO extracted from the fresh rhizomes of *A. galanga* were analyzed. AGO and its three components of interest, 1,8-cineole, 4-allylphenyl acetate, and methyl eugenol were selected to investigated possible pathways of anesthetic action in mammals. For this purpose, the binding of test specimens to GABA_A_ receptors in rat cortical membranes was studied using the [^3^H]muscimol binding assay and an in silico modeling platform. In addition, a nanoemulsion with AGO (NE-AGO) was formulated to reduce the amount of organic solvent used for dissolving AGO and to determine its potential on AGO delivery. The toxicity of AGO on normal human cells was also investigated for possible use in humans.

## 2. Materials and Methods

### 2.1. Materials

Fresh rhizomes of *A. galanga* were collected from the medicinal plant garden of Chiang Mai University, Chiang Mai, Thailand in February 2018 according to the WHO Guidelines on Good Agricultural and Collection Practices (GACP) for Medicinal Plants. The plant was identified by Wannaree Charoensup (a botanist, Department of Pharmaceutical Sciences, Faculty of Pharmacy, Chiang Mai University, Chiang Mai, Thailand), and the voucher specimen (no. 009245) was deposited at the Herbarium of the Northern Research Center for Medicinal Plants, Faculty of Pharmacy, Chiang Mai University, Thailand.

Fetal bovine serum (FBS), penicillin, streptomycin, L-glutamine, and RPMI 1640 were purchased from GIBCO InvitrogenTM (Waltham, MA, USA). Lymphoprep and 4-allylphenyl acetate were purchased from Axis-Shield PoC AS (Oslo, Norway) and ABCR GmbH (Karlsruhe, Germany). Methyl eugenol, 3-(4,5-Dimethylthiazol-2-yl)-2,5-diphenyltetrazolium bromide (MTT) and DMSO were purchased from Sigma-Aldrich (St Louis, MO, USA). 1,8-cineole, polyoxyethylene sorbitan monooleate (Tween 80), phosphate buffer solution (PBS), and dichloromethane were of analytical grade and were supplied by Merck Millipore (Darmstadt, Germany). Diazepam and [^3^H]muscimol (36.6 Ci/mmol) were obtained from Nycomed Danmark A/S (Hobro, Denmark) and Perkin Elmer (Boston, MA, USA).

### 2.2. Extraction and Chemical Analysis of AGO

Fresh rhizomes of *A. galanga* were washed with clean water and were cut into small pieces before being subjected to hydro-distillation for 3 h. The obtained AGO was analyzed for their chemical compositions by gas chromatography–mass spectrometry (GC–MS) on an Agilent 6890 gas chromatograph coupled to electron impact (EI, 70 eV) using a Hewlett Packard (HP) mass selective detector (MSD), model HP 5973-MSD (Agilent Technologies Inc., Willmington, DE, USA). The HP5-MSI column with a 30.0 m × 0.25 mm internal diameter and a 0.25 mm film thickness (Agilent Technologies Inc., Santa Clara, CA, USA) was used as a capillary column. The analytical conditions were modified from previous studies [22]. Briefly, AGO was diluted with dichloromethane to 1:100 (v/v) and 1 μL of this mixture was injected into GC–MS. The injection and detector temperatures were 250 °C and 280 °C, respectively. The oven temperature was 70 °C. The sample was held isothermally for 3 min and the temperature was then increased by 3 °C/min to 188 °C, and then by 20 °C/min to 280 °C, followed by holding for 3 min. Helium was used as the carrier gas at a flow rate of 1 mL/min. The experiments were performed in triplicate.

### 2.3. Preparation and Characterization of NE-AGO

A stable nanoformulation of NE-AGO, composed of 20% w/w AGO, 10% w/w Tween 80, and 70% w/w water was prepared according to the method previously described [4]. Briefly, the aqueous phase, containing Tween 80 and water, was mixed using a vortex mixer for 5 min, then added to the oil phase composed of AGO. The mixture was stirred at 50 °C for 5 min before being subjected to a high-speed stirring of 16,000 rpm using an Ultra-Turrax T25 (Janke and Kunkel GmbH, Staufen, Germany) for 5 min and passed through a high-pressure homogenizer (Avestin Inc., Ottawa, Canada) under a pressure of 10,000 psi for 7 cycles at room temperature.

The droplet size, size distribution, and zeta potential of the obtained NE-AGO were determined using dynamic light scattering by photon correlation spectroscopy (Zetasizer Nano ZS, Malvern Instruments Ltd., Malvern, UK). The polydispersity index (PDI) value indicates the width of the size distribution. NE-AGO was diluted (1:100 v/v) with purified water to have a suitable scattering intensity before measurement. The results were obtained by averaging at least ten measurements at a fixed angle of 173° at 25 °C. At least three experiments were conducted independently.

### 2.4. [^3^H]Muscimol Binding Assay

Rat brain cortical synaptosomes from adult male Sprague-Dawley rats were prepared according to the method previously described [23]. All animal experiments were carried out in accordance with the European Communities Council Directive (2010/63/EU), as well as the ARRIVE guidelines for the care and use of laboratory animals and the Danish legislation regulating animal experiments. The modulation of [^3^H]muscimol binding to the rat brain cortical homogenate of DMSO solutions containing AGO, or the selected compounds, were compared. DMSO was used as a vehicle control. On the day of the test, the membranes were quickly thawed in the binding buffer (50 mM Tris-HCl buffer; pH 7.4), then homogenized and washed three times by centrifugation (48,000× *g* at 4 °C). The [^3^H]muscimol binding assay was performed in a 96-well format, as previously described [23,24]. For this purpose, aliquots of membrane preparation (75–100 µg protein/aliquot) were incubated with a test substance and with a radioligand [^3^H]muscimol (5 nM) in a total volume of 250 µL at 0 °C for 60 min. Nonspecific binding was determined in the presence of 1 mM GABA, whereas 100 µM of diazepam was used as a control for positive modulation. After incubation for 1 h at 0–4 °C, the binding reaction was terminated by rapid filtration through GF/C unifilters (PerkinElmer, Waltham, MA, USA) using a 96-well Packard FilterMate cell harvester, followed by three successive washes with an ice-cold binding buffer, the addition of the MicroScint-O scintillation fluid (PerkinElmer, Groningen, The Netherlands), and the quantification of the filter-bound radioactivity in a Packard TopCount microplate scintillation counter. The experiments were performed in triplicate and were repeated in at least three independent experiments. Data analyses were performed using GraphPad Prism 7.0b (GraphPad Software Inc., La Jolla, CA, USA). Counts per min values were converted to specific binding by subtracting non-specific binding. For modulation curves, data were fitted by a non-linear regression analysis using the equation for a sigmoidal concentration-response with a variable slope, according to Equation (1):

Y = Bottom + (Top − Bottom)/1 + 10^(logIC^^50 − X) × Hill-Slope^, (1)

where is Y is the response, X is the logarithm of the concentration, and Top and Bottom are the plateaus in the same units as Y. IC_50_ is half the maximal inhibitory concentrations, and log IC_50_ is the concentration giving a response halfway between Bottom and Top. The Hill-Slope is the steepness of the curve. All data were determined in triplicate and repeated in at least three independent experiments.

### 2.5. Computational Method

#### 2.5.1. Preparation of Ligands for Molecular Docking

The three components of AGO, methyl eugenol, 1,8-cineole, 4-allylphenyl acetate, and the positive control, diazepam, were used as docking ligands. The 3D molecular structures of these ligands were downloaded from PubChem [25]. ChemBio3D ultra 11.0 and AutoDockTools were used to minimize the molecular energy and add the Gasteiger partial atomic charges, respectively.

#### 2.5.2. Molecular Docking and Dynamics Simulation

The Lamarckian genetic algorithm (LGA) by the AutoDock program was employed to generate the GABA_A_ receptor–ligand models. A grid was set to cap the upper part of the GABA_A_ receptor (PDB: 6X3T) with 126 × 126 × 126 Å spaced 0.375 Å. Two hundred GA runs were set. A tolerance of 1.0 Å root-mean-square deviation (RMSD) was set for conformational clustering. A docking pose of each compound in the highest populated cluster showing the lowest docking score (kcal/mol) was selected as the candidate pose for each receptor–ligand binding model.

The molecular dynamics simulations of GABA_A_ receptor–ligand complexes were performed with AMBER18 using the PMEMD dynamics engine. The docking models of the GABA_A_ receptor and the ligands methyl eugenol, 1,8-cineole, 4-allylphenyl acetate, and diazepam from molecular docking were added the force field ff03.r1 [26] for the entire simulated systems. Force field parameters for the compounds were generated by Antechamber. The topology and coordinate parameters for each model were generated with tLeap. The complex was solvated in a truncated octahedral periodic box (TIP3P water model). Twenty Cl^−^ ions were added to neutralize the system. The whole systems were successively minimized, heated, and equilibrated. Twenty picoseconds (ps) of the NVT method and 60 ps of the same method were used in the heating stage and equilibrating stage, respectively. Thirty thousand ps of NPT production, run with 300 Kelvin and 1 atm, was carried out to originate a dynamical model of the GABA_A_ receptor and the ligands. A stability parameter of the dynamical GABA_A_ receptor–ligand model of each complex was represented by RMSD. The RMSD plots of the whole complex models, and ligands only, were analyzed. The energetic parameter (binding free energy/binding affinity) was represented by the Molecular Mechanics Generalized Born Surface Area (MM-GBSA) energy. The energy was calculated using the MMPBSA.py script for the 30,000 ps trajectory (representative 150 frames).

### 2.6. Cytotoxicity on Peripheral Blood Mononuclear Cells

The cytotoxicity of AGO and blank nanoemulsion (nanoemulsion without AGO) on normal cells was studied using normal human peripheral blood mononuclear cells (PBMCs). An MTT assay was used according to the previous report, with some modifications [27,28]. The study was approved by the Ethics Committee, Faculty of Associated Medical Sciences, Chiang Mai University (No. AMSEC-64EM-002). Briefly, blood samples were collected by venipuncture from healthy volunteers and were transferred into 15 mL of heparin-coated test tubes. Blood was diluted at a 1:1 ratio (v/v) with 0.1 M PBS, and was layered onto Lymphoprep at a volume ratio of 3:1. PBMCs were collected after centrifugation at 1000× *g* for 30 min and were then washed three times with PBS. The PBMCs were resuspended in a complete RPMI 1640 culture medium supplemented with 10% FBS, 100 unit/mL of penicillin, 100 µg/mL of streptomycin, and 1 mM of L-glutamine. The PBMCs were seeded in a 96-well tissue culture plate (1 × 10^5^ cells/well) and incubated at 37 °C, 5% CO_2_ atmosphere, and 95% relative humidity for 24 h. Meanwhile, the stock solutions of AGO and blank DMSO were added to a complete RPMI 1640 culture medium (100 µL) to achieve final sample concentrations in the range of 15–500 µg/mL. After cell incubation, the obtained sample mixtures were added into each well and further incubated for 1, 3, 6, 12, 24, and 48 h for AGO, and 48 h for the blank nanoemulsion. A mixture composed of 0.5% v/v DMSO in a complete RPMI 1640 culture medium was used as a vehicle control. A MTT stock dye solution (5 mg/mL MTT dye in PBS) was added to each well (15 µL) after the removal of 100 µL of the medium, and the plate was further incubated at 37 °C in a 5% CO_2_ atmosphere. After 4 h, the supernatant was removed, followed by the addition of DMSO (200 µL) to each well, and was mixed thoroughly to dissolve the dye crystals. Absorbance was measured using an AccuReaderTM M965/965+ microplate reader (Metertech Inc., Taipei, Taiwan) at 570 nm with a reference wavelength of 630 nm. All experiments were performed in triplicate and at least three independent experiments confirmed the data. The percent of cell viability was calculated using Equation (2):

% Cell viability = (MA_test_/MA_control_) × 100, (2)

where MA_test_ and MA_control_ are mean absorbance in test wells and mean absorbance in vehicle control wells.

### 2.7. Statistical Analysis

The data are presented as mean ± standard error of the mean (SEM). A statistical evaluation of cytotoxicity study was performed by a one-way ANOVA, followed by Tukey’s post-hoc test, or Dunnett’s *t*-test, where *p* < 0.05 was indicated significant differences. Data was analyzed by using SPSS Statistics for Windows, Version 22.0 (IBM, Armonk, NY, USA).

## 3. Results and Discussion

### 3.1. Extraction and Chemical Analysis of AGO

**Figure 2:**
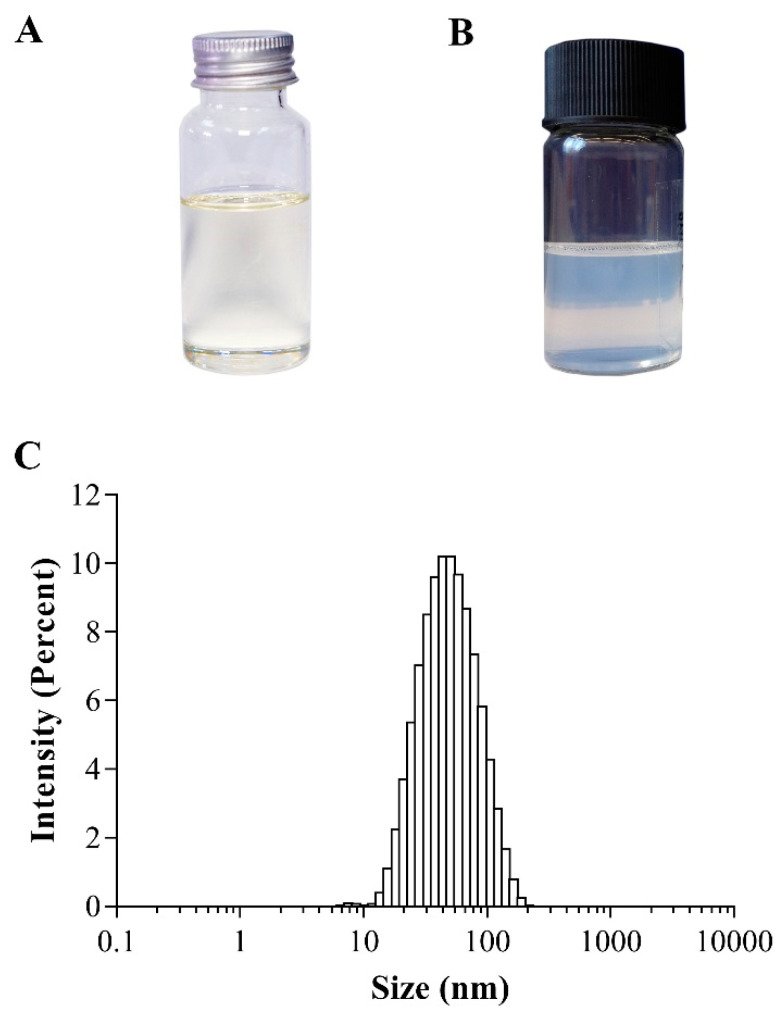
The outer appearance of (**A**) AGO and (**B**) NE-AGO, and (**C**) size distributions of NE-AGO.

AGO appeared as a clear pale yellowish liquid (Figure 2A). The chromatograms of AGO obtained from GC–MS demonstrated 14 identifiable chemical components that represented 97.93% of the total chemical components of AGO (Table 1). The main common components of AGO that have been intensively reported in anesthetic activity are 1,8-cineole, 4-allylphenyl acetate, and methyl eugenol [29,30]. The quantity of these compounds found in AGO in the present study are 41.94 ± 0.13%, 35.70 ± 0.14%, and 3.23 ± 0.02%, respectively. The identified compounds and their quantities are similar to what we have previously reported [5]. Eucalyptol or 1,8-cineole was found to be a major component in AGO. This result is consistent with reports from other groups [31]. However, some differences have been identified in the type and amount of minor compounds. These differences may be due to differences in topography, the post-harvest period, and the post-harvest aeration period [32].

### 3.2. Preparation and Characterization of NE-AGO

As AGO is water immiscible, DMSO is always used to dissolve AGO and enhance water miscibility. However, DMSO shows several disadvantages, due to its toxic effects to animals and humans, and its binding interferences in many biological assays. Nanoemulsions can be prepared without the use of organic solvents [33]. Th eincorporation of water-insoluble substances into the o/w nanoemulsions can improve their water miscibility [34]. In the present study, NE-AGO was formulated without the use of an organic solvent to improve the aqueous miscibility of AGO and to avoid any binding interference of DMSO in the employed binding assay. It was found that the formulated NE-AGO, containing 20% w/w AGO, appeared as a stable translucent o/w nanoemulsion with a white-bluish color, and it showed no phase separation of NE-AGO into two immiscible liquids (Figure 2B). After adding water to NE-AGO for a 100-fold dilution, a rapid dispersion was observed in less than 5 min. In addition, small droplets of AGO, approximately 49 ± 2 nm, with a size distribution, expressed as the polydispersity index (PDI), of 0.24 ± 0.01 were obtained (Figure 2C). Such a low PDI value indicates that the obtained NE-AGO possessed a narrow droplet size distribution. The zeta potential of NE-AGO was negative, with a value of −14.4 ± 0.6 mV. The adsorption of hydroxyl ions in the aqueous system onto the surface of the droplets may lead to slightly negative zeta potential values, and may be the explanation behind the negative zeta-potential of this formulation [35,36]. A high zeta potential of the nanoemulsions, consisting of ionic surfactants, can indicate their physical stability but not for those consisting of non-ionic surfactants. The nanoemulsions containing non-ionic surfactants can be stabilized by several factors, including the effects of steric hinderance, composition, and emulsifiers. Suitable emulsifier combinations create an elastic interface between two immiscible liquids and effectively suspend the dispersed phase in the dispersion medium in the form of tiny droplets. These droplets are elastic and withstand high degree of tension during deformation. The tiny droplets with uniform size distributions retain high level of stability, are not affected by gravity and they can be suspended in the dispersion medium.

### 3.3. [^3^H]Muscimol Binding

**Figure 3:**
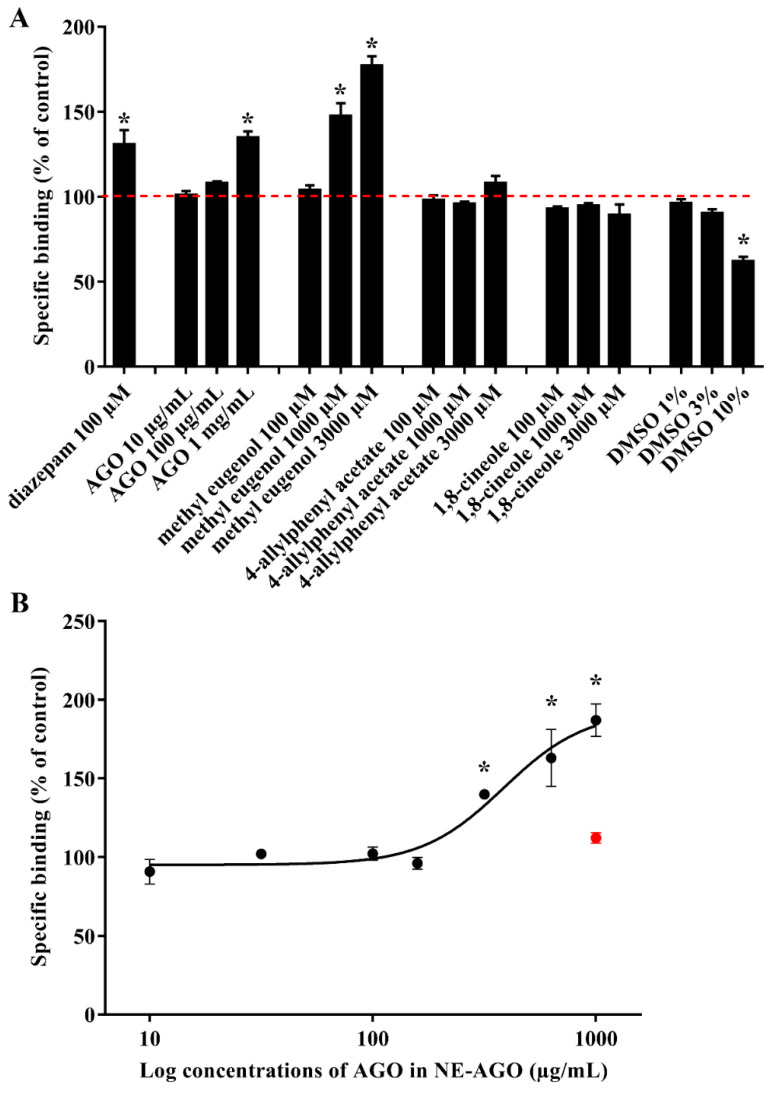
(**A**) Modulation of [^3^H]muscimol binding to rat brain cortical homogenate. The 100% binding (dashed red line) denotes the total specific binding by 30 nM [^3^H]muscimol. (**B**) Concentration-dependent modulation by 20% NE-AGO. Data are presented as mean percentages of specific binding ± SEM. Red point indicates the blank nanoemulsion at the highest concentration. Asterisk (*) indicates significant differences between groups with 100% binding (*p* < 0.05).

[^3^H]Muscimol was previously reported as a high-affinity radioligand to label GABA_A_ receptors in a synaptosomally enriched cortical homogenate preparation from Sprague-Dawley rats [37]. Diazepam was used as a positive control because of its ability to modulate [^3^H]muscimol, as previously reported [38]. It was found that AGO, at a concentration of 1 mg/mL in DMSO, showed a specific modulation of [^3^H]muscimol, which was 130% similar to diazepam, at a concentration of 100 μM, as seen in Figure 3A.

These binding effects indicate that AGO contains constituents that can modulate [^3^H]muscimol binding, and the results prompt further studies. These involved testing the three main constituents of AGO, 1,8-cineole, 4-allylphenyl acetate, and methyl eugenol. The results indicated that only methyl eugenol was a modulator. A specific modulate [^3^H]muscimol binding of AGO and methyl eugenol was according to the different concentrations used in the test. The 1 mg/mL of AGO was equivalent to 2718.98 µM of 1,8-cineole, 2025.99 µM of 4-allylphenyl acetate, and 181.23 µM of methyl eugenol. Moreover, the significance of specific binding depended on methyl eugenol, whereas 1,8-cineole and 4-allylphenyl acetate did not show any specific binding at a concentration range of 100–3000 µM. Although 100 µM of methyl eugenol did not show any specific binding, but increased the concentration of methyl eugenol to 181.23 µM, the compound might be able to exhibit this specific binding. In this study, DMSO was used for dissolving AGO and the three components; therefore, DMSO was also tested separately. The final concentrations of DMSO in AGO and all three components at low, medium, and high concentrations were 1%, 3%, and 10%, respectively. Results clearly showed that DMSO, particularly at concentrations above 3% w/w, significantly decreased the specific binding of [^3^H]muscimol and limited the accuracy of compound testing above 3000 µM. Although DMSO interfered with AGO and the methyl eugenol binding assay, a significant increase in the binding effect can be observed. The binding increased with increasing concentrations of both AGO and methyl eugenol. The result in the system without DMSO, as shown in Figure 3B, shows that as the concentration of NE-AGO increases, the binding also increases, confirming the concentration-dependent binding effect of AGO and methyl eugenol. AGO in NE-AGO, at approximately 500 µg/mL, shows significantly high binding levels.

From these results, it is obviously seen that DMSO interferes with the binding assay. The binding effect of the test samples will be more significant if DMSO was not used as a solvent. Thus, an accurate assessment of the median effective concentration (EC_50_) could not be obtained from the oil or methyl eugenol containing DMSO. Moreover, DMSO at a concentration above 1% w/w dissolved the membrane, while the nanoemulsion did not. To improve aqueous solubility and to exclude the use of DMSO, NE-AGO was used for testing instead. The binding effect of the blank nanoemulsion, as a negative control at the highest tested concentration, was not significantly different from the 100% binding, indicating that the blank itself did not significantly modulate the binding level. The potent agonist value (pEC_50_) can be obtained from the negative logarithm of the EC_50_. The results demonstrate that the EC_50_ value, the mean potent agonist value (pEC_50_ ± SEM), and the maximum binding level expressed as the mean ± SEM of AGO can be obtained in the NE-AGO formulation described, and was found to be 391 µg/mL, 3.41 ± 0.02, and 179 ± 10% of the control, respectively.

### 3.4. Molecular Docking and Dynamics Simulation

**Figure 4:**
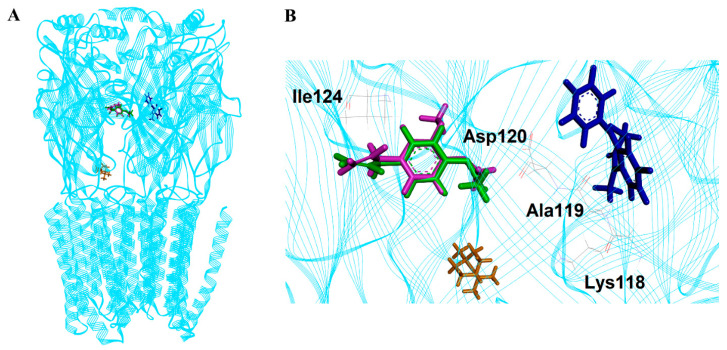
(**A**) The binding locations of the ligands; methyl eugenol (pink), 1,8-cineole (orange), 4-allylphenyl acetate (green), and diazepam (blue) on GABA_A_ receptor (light blue); (**B**) closed view of the docking compounds at the binding locations and the adjacent amino acid residues of the E chain.

The selected poses of all compounds on the GABA_A_ receptor are shown in Figure 4. Methyl eugenol, 4-allylphenyl acetate, and a positive control diazepam bind to the GABA_A_ receptor on the same region that was previously identified as the high-affinity benzodiazepine site (α+/γ-) [39]. On the other hand, 1,8-cineole binds to the different region.

**Figure 5:**
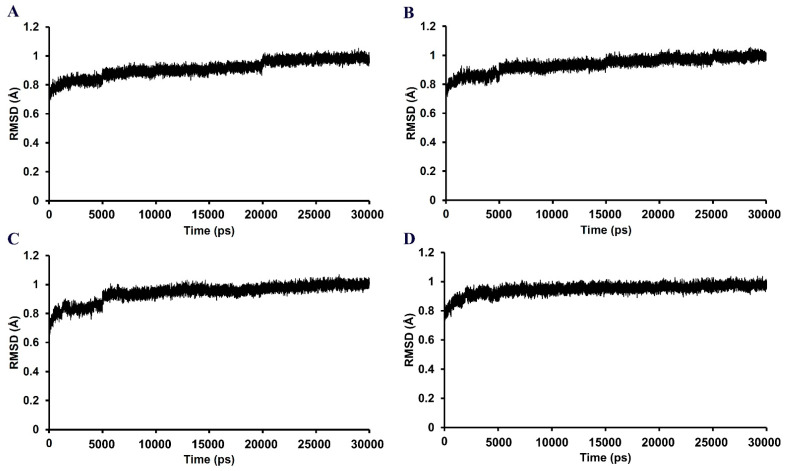
RMSD plots all over the 30,000 ps simulation of the GABA_A_ receptor–ligand coordinates from the (**A**) methyl eugenol, (**B**) 1,8-cineole, (**C**) 4-allylphenyl acetate, and (**D**) diazepam models.

**Figure 6:**
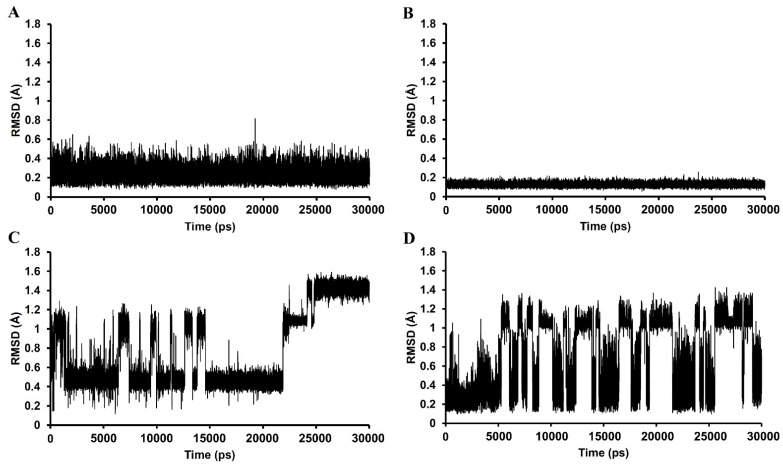
RMSD plots all over the 30,000 ps simulation of the ligand coordinates from the (**A**) methyl eugenol, (**B**) 1,8-cineole, (**C**) 4-allylphenyl acetate, and (**D**) diazepam models.

In the 30,000 ps simulation of the GABA_A_ receptor–ligand binding model, the entire structures of the GABA_A_ receptor complexes with methyl eugenol, 1,8-cineole, 4-allylphenyl acetate, and diazepam remained relatively stable, as shown in Figure 5. The overall system of all ligand structures showed a stable RMSD pattern throughout the 30,000 ps simulations, indicating a high stability of the complexes. However, considering the RMSD plot of each ligand structure, as shown in Figure 6, the result clearly indicated that the molecular dynamics simulation of only the GABA_A_ receptor–methyl eugenol complex provided a stable structure of methyl eugenol that were all over 30,000 ps. The convergence behavior of the complex was confirmed by its MM-GBSA energy of −22.16 kcal/mol, as shown in Table 2, which was significantly more negative than the positive control (−15.92 kcal/mol). This implies that the binding interaction of methyl eugenol is stronger than diazepam, specified by the MM-GBSA energy and a less stable RMSD pattern of diazepam. Regarding the binding model of 1,8-cineole, the ligand structure exhibited a low rearrangement, which was lower than that of the methyl eugenol structure. However, when considering the MM-GBSA energy of the 1,8-cineole binding model, the energy was less negative than that of methyl eugenol and 4-allylphenyl acetate. This could be due to high rigidity of its structure that caused it to be difficult to rotate the groups, as well as a lack of interacting moiety. Even though the energy of 1,8-cineole is close to diazepam, but the binding site of 1,8-cineole is quite far from diazepam. This indicates that a lack of a binding position to the diazepam binding site of 1,8-cineole may negate the potential of this compound [40]. In the 4-allylphenyl acetate binding model, although its binding affinity, represented by the MM-GBSA energy, was more negative than diazepam, the ligand structure showed highly unstable RMSDs, which were all over 30,000 ps (≥1 Å difference). This instability can result in an unstable affinity and a subsequent inactivity of the compound. The results of the in silico model support the in vitro results, with only methyl eugenol showing positive GABA_A_ receptor modulation at the highest concentration, while 1,8-cineole and 4-allylphenyl acetate were inactive.

### 3.5. Cytotoxicity to Human Normal Cells

**Figure 7:**
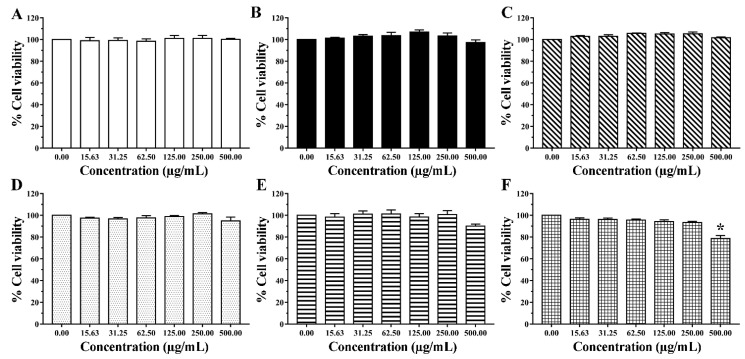
Dose-response curves of viability of PBMCs exposed to AGO in DMSO for (**A**) 1 h, (**B**) 3 h, (**C**) 6 h, (**D**) 12 h, (**E**) 24 h, and (**F**) 48 h. Data are presented as means ± SEM and were analyzed based on a one-way ANOVA followed by Tukey’s post-hoc test (*p* < 0.05). Asterisk (*) indicates significant differences between concentrations of AGO.

PBMCs of normal volunteers are commonly used as model cells for the inhibitory evaluation of the test samples on the cell proliferation of normal human cells [28,41]. In the present study, AGO and the blank nanoemulsion were tested for safety issues in PBMCs. The dose-response curves showed that PBMCs had a high (>80%) survival after exposure to AGO over the entire exposure and concentration ranges, as seen in Figure 7. PBMC viability values above 80% are generally considered safe for use in humans [42]. The results showed that the IC_50_ value of AGO at the total exposure time was greater than 500 µg/mL. In addition, there were no significant differences in AGO concentrations and exposure times to PBMC survival rates in the groups exposed to AGO for 1, 3, 6, 12, and 24 h (*p* < 0.05), except for 48 h, where the AGO toxicity against PBMCs tended to depend on the AGO concentration. However, high PBMC survival was observed to be approximately 80% (78.65 ± 2.83%), even when exposed to concentrations of AGO as high as 500 µg/mL. These results indicated that AGO was nontoxic to PBMCs. The cell viability was greater than 90% when the blank nanoemulsion at a final concentration of 500 µg/mL was used, indicating that the blank nanoemulsion was also nontoxic to PBMCs.

Methyl eugenol, 4-allylphenyl acetate, and 1,8-cineole are the active components in AGO. The results from the cytotoxicity test suggested that the viability of the cells is found to be higher than 80% after exposure to AGO. Therefore, this plant can be considered as safe for humans. The modulation efficiency due to AGO is related to the methyl eugenol content in AGO. Computational docking and dynamics simulations into the GABA_A_ receptor complex model showed the same region binding site of benzodiazepine, methyl eugenol, and 4-allylphenyl acetate. However, the GABA_A_ receptor complex of methyl eugenol is significantly more stable than that of 4-allylphenyl acetate. In addition, the NE-AGO, containing 20% w/w AGO, that was formulated without the use of organic solvents to improve the aqueous miscibility of AGO, and to avoid any binding interference by DMSO in the employed binding assay, appeared as a stable translucent o/w nanoemulsion with white-bluish color. NE-AGO possessed a small droplet size (approximately 50 nm) and a narrow droplet size distribution (PDI = 0.24 ± 0.01). NE-AGO showed a significant increase in specific [^3^H]muscimol binding with EC_50_ of 391 µg/mL.

Anesthesia can be induced through many pathways, including enhancing inhibitory signals or blocking excitatory signals. Generally, anesthetics act as GABA_A_ receptor agonists, N-methyl-D-aspartate receptor antagonists, α2-Adrenoceptor agonists, or dopaminergic receptor antagonists. In the present study, GABA_A_ receptors were emphasized because they are the most common targets for human anesthetics. The current study indicates that one of the key components for the anesthetic activity of AGO is methyl eugenol, whose potential pathway of action is the interaction with the GABA_A_ receptors. Our results on the anesthetic pathway of methyl eugenol is in good agreement with the previous report [29]. There was also no relationship between the constituent content and the mechanism of action. However, the EC_50_ and maximal binding levels of the NE-AGO formulation correlates well to the measured content of methyl eugenol present in this essential oil (180 µM). The results confirmed that AGO anesthesia was caused by methyl eugenol through the involvement of GABA_A_ receptors.

In addition, 1,8-cineole has been reported to directly affect Na^+^ channels of the superior cervical ganglion neurons that are likely to be the major cause of the excitability blockade [43]. Our results supported this previous finding that the major pathway of 1,8-cineole is not by binding to GABA_A_ receptors. Furthermore, the mechanism of action of some local anesthetics, such as lidocaine and procaine, has been reported via the Na^+^ channels blockade pathway [39,44]. The blockade of excitability, caused by 1,8-cineole, appears to be identical to these classic local anesthetics. Even though nanoemulsion showed its suitability to deliver AGO, further studies are required to determine whether the pharmacokinetic characteristics of AGO and its three main constituents, 1,8-cineole, 4-allylphenyl acetate, and methyl eugenol, will influence the acceptability in using this essential oil as a new local anesthetic in humans.

## 4. Conclusions

The present study demonstrates that AGO can be easily obtained by the hydrodistillation of fresh rhizomes of *A. galanga*. A composition analysis of AGO, using GC–MS, shows that methyl eugenol, 1,8-cineole, and 4-allylphenyl acetate are the key components in AGO. The binding of AGO and these compounds to the GABA_A_ receptors of rat cortical membranes using the [^3^H]muscimol binding assay reveals that only AGO and methyl eugenol can modulate [^3^H]muscimol. The modulation efficiency, due to AGO, is related to the methyl eugenol content in AGO. The in silico modeling platform indicates that methyl eugenol and 4-allylphenyl acetate can bind to GABA_A_ receptors on the same region as the high-affinity benzodiazepine site; however, the GABA_A_ receptor complex of methyl eugenol is significantly more stable than that of 4-allylphenyl acetate. It is concluded that one of the mechanisms of the anesthetic action of AGO in mammals is due to methyl eugenol, through the GABA_A_ receptor modulation pathway. The other two components, 1,8-cineole and 4-allylphenyl acetate, did not show GABA_A_ receptor modulation, and their anesthetic action might instead be related to other mechanisms. In addition, to reduce the toxicity and binding interferences in the [^3^H]muscimol biological assay caused by DMSO, the use of blank nanoemulsions, instead of DMSO, for enhancing the water miscibility of AGO is suggested. AGO and the blank nanoemulsion are well-tolerated by human normal cells. Therefore, NE-AGO is suggested to be useful for further studies in humans.

## Figures and Tables

**Table 1:** Chemical constituents in AGO.

No.	Components	Retention Time (min)	Amount (%)
1	β-Pinene	5.08	0.72 ± 0.07
2	1,8-Cineole	6.62	41.94 ± 0.13
3	α-Terpineol	11.49	2.64 ± 0.06
4	Terpinen-4-ol	11.89	3.07 ± 0.04
5	Chavicol	15.85	1.33 ± 0.08
6	4-Allylphenyl acetate	19.02	35.70 ± 0.14
7	Geranyl acetate	20.40	0.55 ± 0.01
8	Methyl eugenol	21.39	3.23 ± 0.02
9	α-Farnesene	22.32	0.58 ± 0.05
10	β-Bisaboloene	25.29	0.78 ± 0.01
11	β-Sesquiphellandrene	25.87	0.53 ± 0.01
12	Eugenyl acetate	26.22	1.19 ± 0.02
13	9-Octadecenoic acid	44.86	2.34 ± 0.12
14	9-Octadecenamide	46.53	3.39 ± 0.07
Total			97.99 ± 0.34

**Table 2:** Interaction of GABA_A_ receptor and the ligands. MM-GBSA energies of all models are listed.

Ligand	MM-GBSA Binding Energy (kcal/mol)
Methyl eugenol	−22.16
1,8-Cineole	−16.63
4-Allylphenyl acetate	−23.72
Diazepam	−15.92

## Data Availability

All data are available upon request.
